# Benign Skin Neoplasms among the Histopathological Specimens of Skin Neoplasm in a Teaching Hospital: A Descriptive Cross-sectional Study

**DOI:** 10.31729/jnma.6086

**Published:** 2021-11-30

**Authors:** Sushma Thapa, Arnab Ghosh, Dilasma Ghartimagar, Sudeep Regmi

**Affiliations:** 1 Department of Pathology, Manipal Teaching Hospital, Pokhara, Nepal

**Keywords:** *acrochordon*, *appendageal tumor*, *benign skin neoplasms*, *compound nevus*, *keratinocytic tumor*

## Abstract

**Introduction:**

Skin tumors are relatively uncommon malignancies worldwide, but its incidence has been progressively increased over the last few decades. Skin tumor belongs to a diverse group of neoplasms arising from the epidermis, adnexal structures and dermis rendering the classification difficult. The study aims to find out the prevalence of benign skin neoplasm among the histopathological specimens of skin neoplasm of a teaching hospital.

**Methods:**

A descriptive cross-sectional study among the hospital records of histopathological samples of skin neoplasm in the Department of Pathology of a tertiary care center from January 2017 to December 2020. Ethical approval was taken from the Institutional Review Committee (Ref: MEMG/IRC/427/GA). Convenient sampling was done. Data were entered in Microsoft Excel and analyzed using Statistical Package for the Social Sciences version 21 software. Point estimate at 95% Confidence Interval was calculated with frequency and descriptive statistics.

**Results:**

Out of total skin neoplasm samples, 121 (57.34%) (50.67-64.01 at 95% Confidence Interval) benign skin neoplasms were present. Among them, the majority were keratinocytic tumor 81 (66.9%) followed by skin appendageal 23 (19.0%) and melanocytic tumors 17 (14.0%). Acrochordan 18 (14.9%) and pilomatricoma 12 (9.9%) were the predominant keratinocytic and appendageal neoplasms respectively. Most of the cases occurred in head and neck region 64 (52.9%).

**Conclusions:**

The study concluded that the prevalence of benign skin neoplasm was slightly lower compared to the other studies. Most of the benign skin neoplasms were keratinocytic tumors followed by appendageal and melanocytic tumors. Acrochordan was the commonest benign keratinocytic tumor.

## INTRODUCTION

Skin is the largest organ in the body and forms a protective covering as well as has an endocrine role in synthesizing vitamin D. Histologically, it is composed of several cell types that function interdependently and co-operatively.^1^ The epidermal layer is composed of 90% of keratinocytes and remaining 10% composed of melanocytes, langerhan cells and merkel cells. Epidermal appendages extend from epidermis to dermis comprising specialized cells like follicular epithelial cells, sebaceous cells, cells of eccrine and apocrine glands. Different cell types give rise to different types of skin tumors.^2^

Benign tumors are sometimes confused clinically with malignancy, so, histologic examination is required to establish a definite diagnosis which is the gold standard for accurate diagnosis.^3^ The different histological types of benign skin neoplasms, demographic profiles of patients, site distribution was classified according to World Health Organization (WHO).^4^

The study aims to find out the prevalence of benign skin neoplasm among the histopathological specimens of skin neoplasm of a teaching hospital.

## METHODS

This was a descriptive cross-sectional study among the skin neoplasm, carried out in the Department of Pathology at Manipal Teaching Hospital of Nepal. The study included hospital records from January 2017 to December 2020. Ethical approval was taken from the Institutional Review Committee (IRC) (Reference no. MEMG/IRC/427/GA). The study included all the histopathologically diagnosed cases of skin neoplasm arising from the epidermis along with melanocytic and adnexal tumors. Both incisional and excisional biopsy specimens were included in the study. The mesenchymal tumors, hematolymphoid tumors, neural tumors, cystic lesions and skin secondaries were excluded from the study. Skin biopsies which are non-neoplastic, without adequate demographic profile and where proper sites not mentioned were also excluded from the study.

The sample size was calculated by using formula,

n = Z^2^ × p × q / e^2^

  = (1.96)^2^ × 0.638 × (1-0.638) / (0.07)^2^

  = 181

Where,

n= required sample size,
Z= 1.96 at 95% Confidence Interval (CI),
p= prevalence of benign skin neoplasms taken from a previous study, 63.84%^5^
q= 1-p
e= margin of error, 7%

The calculated sample size was 181. Taking 10% non-response rate the calculated sample size is 198. However, 211 specimen samples were taken for the study.

The specimens were fixed in 10% formalin and sections were taken from the representative areas. The tissues were processed and embedded in paraffin wax. Thin sections of 3-5 microns were made and stained with Hematoxylin and Eosin stain as per the standard protocol. Sections were studied under light microscopy and histological classification of the tumor was done according to WHO classification guidelines of skin tumors (2018). The relevant clinical details of the patients with skin neoplasms diagnosed on histopathology during the study period were retrieved from the departmental data bank.

The data collected were entered in Microsoft Excel sheet and analyzed by using Statistical Package for the Social Sciences (SPSS) version 25 software. Point estimate at 95% CI was calculated.

## RESULTS

During the study period, there were a total 211 cases of skin neoplasms among which 121 (57.34%) (50.6764.01 at 95% Confidence Interval) cases were benign skin neoplasm. Among the benign skin neoplasm, the majority were keratinocytic tumor 81 (66.9%) followed by skin appendageal 23 (19.0%) and melanocytic tumors 17 (14.0%). Overall, females 72 (59.5%) were predominantly affected than males 49 (40.5%) with female to male ratio being 1.5:1. Benign tumors were observed in all the age groups ranging from 10 years to 99 years with the mean of 47.35±19.716 years. Majority of the neoplasms were encountered in the third and sixth decade of life with 22 (18.2%) cases each. (Table 1) The benign keratinocytic tumors were encountered mainly in the 61-70 years of age group 18 (22.2%) whereas the appendageal tumors and melanocytic tumors were commonly seen in the age group of 51-60 years 5 (21.5%) and 21-30 years 7 (41.2%) respectively (Table 1).

**Table 1:** Age group and gender distribution of benign skin neoplasms (n=121).

Age Group (Years)	Nature of Lesion						Total n (%)
	Keratinocytic		Appendageal		Melanocytic		
	Male n (%)	Female n (%)	Male n (%)	Female n (%)	Male n (%)	Female n (%)	
0 - 10	0	0	1 (4.3)	0	0	0	1 (0.8)
11 - 20	3 (3.7)	1 (1.2)	1 (4.3)	0 (0.0)	1 (6.0)	3 (17.6)	9 (7.4)
21 - 30	4 (4.9)	7 (8.6)	1 (4.3)	2 (8.7)	2 (11.8)	5 (29.4)	21 (17.4)
31 - 40	6 (7.4)	9 (11.1)	1 (4.3)	3 (13.0)	0	3 (17.6)	22 (18.2)
41 - 50	3 (3.7)	6 (7.4)	2 (8.7)	1(4.3)	0	2 (11.8)	14 (11.6)
51 - 60	3 (3.7)	8 (9.9)	2 (8.7)	3 (13.0)	0	1 (6.0)	17 (14.0)
61 - 70	11 (13.6)	7 (8.6)	1 (4.3)	3 (13.0)	0	0	22 (18.2)
71 - 80	5 (6.2)	4 (5.0)	0	1 (4.3)	0	0	10 (8.3)
> 80	2 (2.5)	2 (2.5)	0	1 (4.3)	0	0	5 (4.1)
	37 (45.7)	44 (54.3)	9 (39.1)	14 (60.9)	3 (17.6)	14 (82.4)	121 (100.0)

In the current study, benign skin neoplasms were most commonly encountered in the head and neck region 64 (52.9%) followed by lower extremities 19 (15.7%) and upper extremities 14 (11.6%). (Table 2)

**Table 2:** Site distribution of various benign skin neoplasms (n=121).

	Nature of Lesion				
		Keratinocytic n (%)	Adnexal n (%)	Melanocytic n (%)	Total n (%)
	Head and Neck region	32 (39.5)	18 (78.3)	14 (82.4)	64 (52.9)
	Lower extremities	16 (19.8)	3 (13.0)	0(0.0)	19 (15.7)
	Upper extremities	12 (14.8)	2 (8.7)	0(0.0)	14 (11.6)
Site	Trunk	9 (11.1)	0 (0.0)	3 (17.6)	12 (9.9)
	Female genital	5 (6.2)	0 (0.0)	0(0.0)	5 (4.1)
	Perianal	5 (6.2)	0 (0.0)	0(0.0)	5 (4.1)
	Breast	2 (2.5)	0 (0.0)	0(0.0)	2 (1.7)
Total		81 (66.9)	23 (19.0)	17 (14.0)	121 (100.0)

Among the keratinocytic tumors, the majority were acrochordon 18 (14.9%) followed by squamous papilloma 17 (14.0%) and verruca vulgaris 16 (13.2%). Pilomatricoma 12 (9.9%) was the predominant appendageal benign neoplasms followed by proliferating trichilemmal cyst 4 (3.3%) (Table 3). Compound nevus 9 (7.4%) was the most frequently observed benign melanocytic tumor followed by intradermal nevus 8 (6.6%) (Table 3).

**Table 3:** Frequency distribution of various benign skin neoplasms (n=121).

Nature	Histopathological Diagnosis	Gender		n (%)
		Male n (%)	Female n (%)	
Keratinocytic	Acrochordon	7 (38.9)	11 (61.1)	18 (14.9)
	Squamous Papilloma	6 (35.3)	11 (64.7)	17 (14.0)
	Verruca Vulgaris	11 (68.8)	5(31.3)	16 (13.2)
	Seborrhoeic Keratosis	5 (33.3)	10 (66.7)	15 (12.4)
	Keratoacanthoma	4 (44.4)	5 (55.6)	9 (7.4)
	Bowen's Disease	2 (50.0)	2 (50.0)	4 (3.3)
	Verruca Plana	2 (100.0)	0 (0.0)	2 (1.7)
Melanocytic				
	Compound Nevus	1 (11.1)	8 (88.9)	9 (7.4)
	Intradermal Nevus	2 (25.0)	6 (75.0)	8 (6.6)
Appendageal				
Eccrine & Apocrine differentiation	Chondroid Syringoma	0 (0.0)	1 (100.0)	1 (0.8)
Follicular differentiation				
	Pilomatricoma	6 (50.0)	6 (50.0)	12 (9.9)
	Proliferating Trichilemmal Cyst	1 (25.0)	3 (75.0)	4 (3.3)
	Trichoepithelioma	0 (0.0)	3 (100.0)	3 (2.5)
	Trichilemmoma	1 (100.0)	0 (0.0)	1 (0.8)
Sebaceous differentiation	Nevus Sebaceous	1 (100.0)	0 (0.0)	1 (0.8)
	Sebaceous Adenoma	0 (0.0)	1 (100.0)	1 (0.8)
Total		49 (40.5)	72 (59.5)	121 (100.0)

There was a female preponderance 11 (61.1%) in acrochordon and were mainly found in the age group of 31-40 years 5 (27.8%) with most common site being the perianal region 4 (22.2%). Pilomatricoma was observed equally in both the male and female with 6 (50.0%) cases each and were mainly seen in the head and neck region 8 (66.7%) with more predilection towards the scalp 5 (41.7%). Among the 17 cases of benign melanocytic tumor, 9 (7.4%) comprised compound nevus with female predominance 8 (88.9%) and majority occurring in the 11-20 years of age (Table 3).

## DISCUSSION

A wide range of benign and malignant tumors are encountered in a clinical practice. There is an increase in the prevalence of skin malignancy in the Nepalese society.^6^ Accurate identification of skin lesion is vital to ensure not to miss the malignancies and that they are treated early to avoid morbidity and mortility.^7^ Skin biopsy is the method to assist the dermatologists to reach a definitive diagnosis and guide patient management. In this study, benign tumors were more common than the malignant tumor comprising of 57.34% comparable to the study done by Shrivastava V, et al. (63.8%), Narhire VV, et al. (69.4%) and Kaur R, et al. (67.27%).^5,8,9^ But the percentage of benign tumors were relatively higher in the studies done by Pappala P, et al. and Sherpa P and KC SR which may be due to difference in the number of cases included, time period of the study and geographical distribution.^10,11^ In contrast to this study, a predominance of malignant tumor were noted in various studies performed by Gundalli S, et al. Nandyal SS, et al. and Samanta M, et al. which may be due to increase number of referral received in higher center as well as geographical variation.^12-14^

In the current study, majority of the cases were keratinocytic tumor 81 (66.9%) followed by skin appendageal 23 (19.0%) and melanocytic tumors 17 (14.0%) similar to the study done by Uplaonkar VS, et al. in which out of 36 cases studied, keratinocytic, appendageal and melanocytic tumor were 41.7%, 38.9% and 19.4% respectively.^15^ In the study done by Pappala P, et al. among the 43 cases, keratinocytic, melanocytic and appendageal tumor were 60.52%, 23.3% and 16.3% respectively.^10^ Similar to our study Bari V, et al. also found keratinocytic tumor as the commonest tumor.^16^ Whereas in the study done by Gundalli S, et al.^12^ Narhire VV, et al.^8^ benign skin adnexal tumor (54.7%) is the commonest tumor encountered whereas other studies found benign melanocytic tumor as the most common tumor.^5,14^

Similar to the current study, other studies also had female predominance in benign skin neoplasms whereas studies performed by Kaur R, et al. and Bari V, et al. concluded that benign neoplasms were more common in males than in females.^3,5,9,16^

The benign skin tumors were seen in all the age groups ranging from 10 to 99 years but majority was encountered in the third decade and sixth decade of life whereas various studies have demonstrated that benign neoplasms are more commonly in the younger age group.^3,9,11,13^

In the current study, majority of the benign keratinocytic tumor were seen in the sixth decade of life (22.2%) in contrast to the study done by Sherpa P and KC SR where third decade was the predominant age group.^11^

This study concluded that head and neck region was the commonest involved site followed by extremities which is comparable to Shrivastava V et al., Narhire VV, et al., Bari V, et al. and Sharma A, et al.^5,8,16,17^ This finding supports the fact that skin neoplasms are the most common in areas of the body with maximum sun exposure.

Among the 81 cases keratinocytic tumor, majority of the cases were acrochordan (14.9%) whereas, Pappala P, et al. observed 21.4% of the cases and Sherpa P and KC SR observed only 4%.^10,11^ Some other studies conducted by Shrivastava V, et al. Kaur R, et al. and Bari V, et al. reported verrucas as the commonest benign neoplasm whereas study done by Pappala P, et al. observed squamous papilloma as the commonest benign skin neoplasm.^5,9,10,16^

Appendageal tumors are neoplasms whose differentiation is towards one or more of the adnexal structures of the skin. In the current study, frequency of occurrence of hair follicular differentiation was the highest (86.9%) followed by sebaceous differentiation (8.7%). In contrast, study done by Gundalli S, et al. observed peak incidence of benign appendageal tumors of sweat gland differentiation (55.17%) followed by hair follicular differentiation (44.83%).^12^ However, in concordance to this study, they also had Pilomatricoma as the predominant benign skin appendageal neoplasm. Similar findings were observed by Shrivastava V, et al., Sherpa P and KC SR.^5,11^

With regard to the benign melanocytic tumor, there were predominance of compound nevus in this study in contrast to the study done by Samantha M, et al. and Sonam SK, et al. where Intradermal nevus is the most common benign melanocytic tumor.^14,18^

The study has its limitations. As it is a descriptive crosssectional study, it is not representing the population at large. Clinical presentations and outcomes of malignant skin neoplasms were not studied. Another limitation of the study was the immunohistochemistry and molecular study which was not present in our hospital set up, so it was not possible to confirm and correlate the histomorphological findings with the underlying genetic mutations leading to skin cancer.

## CONCLUSIONS

The study concluded that the prevalence of benign skin lesions was slightly lower compared to other studies.

Majority of the benign skin neoplasms encountered were keratinocytic tumors followed by appendageal and melanocytic tumors. Acrochordan was the commonest benign keratinocytic tumor. Most of the cases were observed in the head and neck region similar to the other studies.
